# RNA-Seq analysis identifies key genes associated with haustorial development in the root hemiparasite *Santalum album*

**DOI:** 10.3389/fpls.2015.00661

**Published:** 2015-09-01

**Authors:** Xinhua Zhang, Oliver Berkowitz, Jaime A. Teixeira da Silva, Muhan Zhang, Guohua Ma, James Whelan, Jun Duan

**Affiliations:** ^1^Key Laboratory of Plant Resources Conservation and Sustainable Utilization, South China Botanical Garden, Chinese Academy of SciencesGuangzhou, China; ^2^Australian Research Council Centre of Excellence in Plant Energy Biology, The University of Western AustraliaCrawley, WA, Australia; ^3^Department of Botany, Australian Research Council Centre of Excellence in Plant Energy Biology, School of Life Science, La Trobe UniversityBundoora, VIC, Australia; ^4^Independent ResearcherKagawa-ken, Japan

**Keywords:** sandalwood, hemiparasite, haustorium development, RNA sequencing, transcriptome analysis

## Abstract

*Santalum album* (sandalwood) is one of the economically important plant species in the Santalaceae for its production of highly valued perfume oils. Sandalwood is also a hemiparasitic tree that obtains some of its water and simple nutrients by tapping into other plants through haustoria which are highly specialized organs in parasitic angiosperms. However, an understanding of the molecular mechanisms involved in haustorium development is limited. In this study, RNA sequencing (RNA-seq) analyses were performed to identify changes in gene expression and metabolic pathways associated with the development of the *S. album* haustorium. A total of 56,011 non-redundant contigs with a mean contig size of 618 bp were obtained by *de novo* assembly of the transcriptome of haustoria and non-haustorial seedling roots. A substantial number of the identified differentially expressed genes were involved in cell wall metabolism and protein metabolism, as well as mitochondrial electron transport functions. Phytohormone-mediated regulation might play an important role during haustorial development. Especially, auxin signaling is likely to be essential for haustorial initiation, and genes related to cytokinin and gibberellin biosynthesis and metabolism are involved in haustorial development. Our results suggest that genes encoding nodulin-like proteins may be important for haustorial morphogenesis in *S. album*. The obtained sequence data will become a rich resource for future research in this interesting species. This information improves our understanding of haustorium development in root hemiparasitic species and will allow further exploration of the detailed molecular mechanisms underlying plant parasitism.

## Introduction

The Santalaceae is a widely distributed family of flowering plants and its members, like other species of the Santalales, are hemiparasitic plants which can produce photosynthetic products for themselves, but need to obtain water and solutes from the host. *Santalum album*, or the sandalwood tree, is one of the economically important members in this family. It is highly valued due to its aromatic heartwood, which contains sandal oil used in perfumes, cosmetics, medicine, aromatherapy, and more recently in the prevention of skin cancer (Baldovini et al., 2011). In nature, at least 300 species, including *S. album* itself, can act as hosts of sandalwood tree, supplying water and nutrients through a unique organ termed the haustorium, especially during early phases of development (Fineran, 1963; Nagaveni and Vijayalakshmi, 2003).

Approximately 1% of angiosperm species are parasitic (Westwood et al., 2010). Usually, parasitic plants depend on host root-derived chemical signals to induce seed germination and the formation of haustoria. For example, seeds of *Striga* species only germinate in response to compounds from the host-borne root exudate, such as strigolactones (Kubo et al., 2009). Following seed germination, most parasitic species will develop a functional haustorium depending on a second chemical signal also derived from the host exudates, such as 2,6-dimethoxy-*p*-benzoquinone (DMBQ), phenolic acids, and flavonoids which have been identified as haustoria-inducing factors (HIFs) (Cook et al., 1966; Albrecht et al., 1999; Yoneyama et al., 2008). Studies have shown that a single-electron reducing quinone oxidoreductase (TvPirin) was required to induce haustorium development in the roots of *Triphysaria versicolor* (Bandaranayake et al., 2010, 2012). *S. album* is an aggressive root hemiparasitic tree. When its seeds are pretreated with 2–8 mM GA_3_ for 12 h, they can germinate in sand or *in vitro* on Murashige and Skoog medium (Nikam and Barmukh, 2009; Zhang et al., 2012), and then generate haustoria within 30 days from germination without the need for induction by HIFs (Barrett and Fox, 1997). However, little is known about the molecular mechanisms involved in haustorial development in *S. album*.

Plant hormones play important roles in regulating plant developmental processes. Studies have shown that the classical phytohormones auxin, cytokinin (CK), gibberellin (GA), abscisic acid (ABA), jasmonic acid (JA) and ethylene (ET), and new groups of plant hormones, brassinosteroids (BR), and strigolactones (SL) were significantly involved in modulating plant growth and development in higher angiosperms (Durbak et al., 2012; Cheng et al., 2013; Fonseca et al., 2014). Several studies on the involvement of plant hormones in the parasite/host association have been published (Lechowski and Bialczyk, 1996; Jiang et al., 2004, 2005). The localized accumulation of IAA and ET are early events in haustorium development in the hemiparasitic *T. versicolor* (Tomilov et al., 2005). Genes involving polar auxin transport, GA-biosynthetic and metabolic enzymes and SL biosynthetic enzymes, MORE AXILLARY GROWTH (MAX)1, MAX3, and MAX4 were differentially expressed in the infective stages of the shoot parasitic dodder, *Cuscuta pentagona* (Ranjan et al., 2014). Our previous study indicated that endogenous levels of IAA, CK, GA_3_, and ABA were higher in haustoria than in seedling roots of sandalwood (Zhang et al., 2012).

In the last few years, using RNA sequencing (RNA-seq) and *de novo* assembly, transcriptomic analyses revealed the conservation of chlorophyll synthesis in root parasitic Orobanchaceae (Wickett et al., 2011) and host-specific patterns of parasite gene expression at the interface between *T. versicolor* and *Zea mays* or *Medicago truncatula* (Honaas et al., 2013). The transcriptome of the parasitic weed *C. pentagona* showed that genes encoding transporters, which regulate responses to stimuli, as well as genes associated with cell wall modifications, were up-regulated in the process of parasitism (Ranjan et al., 2014). Many parasitic plants can cause severe damage to economically important crops (Spallek et al., 2013; Kaiser et al., 2015). However, there are a few plants that are important sources of medicine and aromatic heartwood, such as *S. album* and *Cistanche deserticola*. To improve the growth of beneficial parasitic plants or to reduce the damage caused by these plants on crops during agricultural cultivation, application of exogenous signaling compounds such as plant hormones could regulate the number of haustoria similar to legumes in which nodule numbers can be increased or decreased by applying GA_3_ (Lievens et al., 2005; Maekawa et al., 2009). Therefore, the phytohormone-mediated regulation of haustorial development of parasitic plants needs further analysis.

In the present study, we constructed a transcriptional profile of haustorial development by RNA-seq, identified genes, and the main pathways involved in haustorium development, and revealed the phytohormone-mediated regulation of haustorium development. This information provides a valuable resource for further exploration and understanding of the detailed molecular mechanisms leading to haustorial development and underlying host-parasite interactions in angiosperms.

## Materials and methods

### Plant materials

Two hundred fully ripe *S. album* seeds were obtained from a 15-year-old sandalwood tree in the South China Botanical Garden in March 2012. The seeds were pretreated with a 2 mM solution of GA_3_ for 12 h at room temperature and then sown in a sand bed at a depth of 2–3 cm in the greenhouse of the South China Botanical Garden. One month later, radicles about 3 cm in length emerged and the germinants were transferred to pots filled with a mixture of peat soil: vermiculite (3: 1, v/v) (10 cm in 2-liter pots). A total of 150 elite *S. album* seedlings were divided into three groups, 50 for pre-attachment haustoria (PrAH), 50 for post-attachment haustoria (PoAH) and 50 for seedling roots (R) as controls. Host plant infection followed Zhang et al. (2012)'s method. Plants were cultivated in a greenhouse with a day/night temperature of 25°C/18°C and 70–80% relative humidity, with a 12-h photoperiod and a photosynthetic photon flux density of 180–260 μmol m^−2^ s^−1^. During the experiment, the pots were fertilized with 10 ml of 5% complex fertilizer for each pot per week, weeded by hand when necessary and watered twice daily with tap water at 8:00 am and 18:00 pm

To obtain an overview of the transcriptome during *S. album* haustorium development, all stages of haustorial development before attachment to the host root were collected from 1 to 10 days after haustorium initiation (around 30–40 days after planting) and mixed as the PrAH sample. A typical haustorium with an inverted conical shape before attachment to the host root is shown in Figure S1A. Infective haustoria before penetrating into the host root were isolated from the host root using a sharp blade at 10–20 days of development (about 40–50 days after planting) (Figure S1B). A small portion of epidermis of the host root sticking out from the contact face of the haustoria with the host root was carefully stripped and removed with small tweezers, and the remaining haustorial tissue was pooled as the PoAH sample. Twenty-day-old non-haustorial seedling roots were collected as the R sample. Haustoria and seedling roots were harvested with 10 individuals pooled per replicate, in which the former consisted of 50 haustoria per sample. In total, three biological replicates per tissue were collected. The samples were quickly frozen in liquid nitrogen (N_2_) after cleaning with sterile water and stored at -80°C until use.

### RNA extraction and sequencing

Total RNA was first extracted from collected tissue according to the column plant RNAout 2.0 extraction kit instructions (TIANDZ, Inc. Beijing, China, with DNase). Then RNA was purified using the Qiagen RNAeasy Mini kit (Qiagen, USA) and quantified using a NanoDrop ND-1000 spectrophotometer (Nanodrop Technologies, USA). The quality of total RNA was determined by an Agilent 2100 Bioanalyzer (Agilent Technologies, Palo Alto, Calif.) and samples having an RNA Integrity Number (RIN) greater than 9.0 were selected for cDNA preparation. Three cDNA libraries were constructed from PrAH, PoAH, and R samples in which equal amounts of total RNA were pooled from three biological replicates per tissue using Illumina's TruSeq RNA sample Prep Kit v2 following the procedures outlined in the manufacturer's manual (Illumina, Inc, San Diego, CA). Briefly, mRNA was separated from total RNA using oligo (dT) magnetic beads (Invitrogen, USA), then fragmented into short fragments using fragmentation buffer (Ambion). After synthesizing first-strand cDNAs using random hexamer primers, the second strand was synthesized using appropriate buffers, dNTPs, RNase H, and DNA polymerase I (Invitrogen, USA). After purification using the QiaQuick PCR extraction kit (Qiagen), double-stranded cDNAs were resolved with EB buffer for end repair and to add single nucleotide A (adenine). Short fragments (200 ± 20 bp) were connected to adapters, suitable fragments were selected for PCR amplification as templates after verification on 2% agarose gel electrophoresis, then an Agilent 2100 Bioanalyzer and ABI StepOnePlus Real-Time PCR Systems (Life Technologies, Grand Island, NY, USA) (TapMan Trobe) were used to quantify and determine the quality of the sample library. These RNA-seq libraries were sequenced in a single lane of a HiSeq 2000 platform at the Beijing Genome Institute (BGI, Shenzhen, China) and reads were generated in a 100 bp paired-end format according to the manufacturer's instructions (Illumina Inc. San Diego, CA). RNA-seq read data has been deposited in the NCBI SRA database under accession SRA150639.

### *De nov*o transcriptome assembly and functional annotation

For the assembly of RNA-seq reads into contigs, the Velvet/Oases pipeline was applied (Schulz et al., 2012). In total, 65,312,243 paired-end reads of the three tissue samples were obtained after filtering reads. These reads were of high quality with a Phred score of above +30 and a minimal length of 90 bases. To optimize Velvet assembly parameters, a Perl-based script was used (VelvetOptimiser Version 2.2.5, Victorian Bioinformatics Consortium, Monash University, Melbourne, Australia) which identified an optimal k-mer length of 59 for the assembly from multiple analyses with varying k-mer lengths. For annotation, homology searches were performed with Blast2go (Conesa et al., 2005) against the NCBI database using the Blastx algorithm with a cut-off of *E* < 10^−15^ and targeted searches using the BLAT algorithm (Kent, 2002) against the *Vitis vinifera* (12x V1 release), Arabidopsis (TAIR10) and poplar (V3 release) transcriptomes. Finally, contigs below 200 bp in length, with no assigned annotation or identified as contamination, were removed from the assembly to yield the *Santalum album* Gene Index Version 1 (SaGI01) with a total of 56,011 contigs. Gene ontology (GO) terms for SaGI01 contigs were assigned based on Arabidopsis (TAIR10) transcriptome release.

### Gene expression and biological pathway analysis

For gene expression analysis, RNA-seq reads for each tissue were mapped to the SaGI01 transcriptome using the Bowtie tool (Langmead et al., 2009). To quantify transcript abundances and identify significant changes in transcript expression, the Cuffdiff tool with an upper quartile normalization and multi-read correction was applied to obtain fragments per kilobase of transcript per million mapped reads (FPKM) values (Trapnell et al., 2010). SaGI01 contigs were classified into MapMan BINs against the Arabidopsis peptide database (Table S1, SAGI_Mapman) and their annotated functions were visualized using the MapMan tool (Thimm et al., 2004).

Important metabolic pathways were further analyzed using the MapMan program based on the differentially expressed genes (DEGs) among the three pairwise comparisons, PrAH vs. R, PoAH vs. PrAH and PoAH vs. R (Table S2). For each of the three analyzed pairwise comparisons, transcript identifiers and the related log2-fold ratios were imported into the MapMan desktop tool (MapMan Site of Analysis^1^). In addition, the MapMan annotation file for the SAGI_Mapman reference set (see above) was imported into this tool. Thus, data were mapped to MapMan BINs, which allowed the visualization of the data in different MapMan pathways. Using the Wilcoxon rank sum test integrated in the MapMan tool, BINs were identified that showed an average BIN response which was significantly different from the response of other BINs, as indicated by their corrected *p*-values in the test (Benjamini Hochberg correction) (Benjamini and Hochberg, 1995).

Hierarchical clustering was performed for DEGs with a fold change ≥8 in at least one pairwise comparison of the three analyzed tissues using the Manhattan distance matrix of the heatmap.2 function in the gplots package of the R environment. Over-representation of GO terms within gene clusters was identified with the BiNGO plugin for Cytoscape using a hypergeometric test after a Benjamini and Hochberg FDR correction with a significance level of *p* < 0.01 (Maere et al., 2005).

### Verification of changes in gene expression using qRT-PCR

To confirm the reliability of the RNA-seq results, qRT-PCR was conducted for 24 DEGs with high or low expression levels. Additionally, the qRT-PCR of 12 genes related to cytokinin and gibberellin biosynthesis and metabolism, and auxin signaling, were tested. Primer sequences are listed in Table S3. Sample collection, total RNA extraction, and purification were followed as described above. The first-strand cDNA was synthesized from 2 μg of total RNA using M-MLV reverse transcriptase (Promega, http://www.promega.com) according to the manufacturer's instructions. The qRT-PCR reactions were performed in 96-well plates using the ABI 7500 fast Real-Time PCR system (Applied Biosystems, USA) and the SYBR® Premix ExTaq™ Kit (Takara, Dalian, China). The specificity of qRT-PCR primers was confirmed by melting curve analyses. Template-less controls for each primer pair were included in each run. The expression level was calculated as 2^−Δ*ΔCt*^ and normalized to the Ct value of *S. album Actin*. The qRT-PCR results were obtained from three biological replicates and three technical repeats for each gene and sample.

## Results

### *De novo* sequence assembly and functional annotation

In total, 65,317,243 high quality paired-end reads were obtained from the three tissue samples after filtering for reads with a Phred score of above +30 and a minimal length of 90 bases *de novo* assembled into 56,011 non-redundant contigs with an average size of 618 bp and a total length of 34,627,134 bp. The length of the contigs ranged from 109 to 8228 bp (Table 1, Figure S2).

**Table 1:** **Summary of *de novo* assembly of RNA-seq data**.

**Parameter**	**Number**
Number of contigs	56,011
Number of contigs in scaffolds	0
Number of contigs not in scaffolds	56,011
Total size of contigs	34,627,134
Longest contig (bp)	8228
Shortest contig (bp)	109
Mean contig size (bp)	618
Median contig size (bp)	352
N50 contig length (bp)	997
L50 contig count	10,277
Contig %A	27.38
Contig %C	22.36
Contig %G	22.7
Contig %T	27.55
Contig %N	0

**Figure 1:**
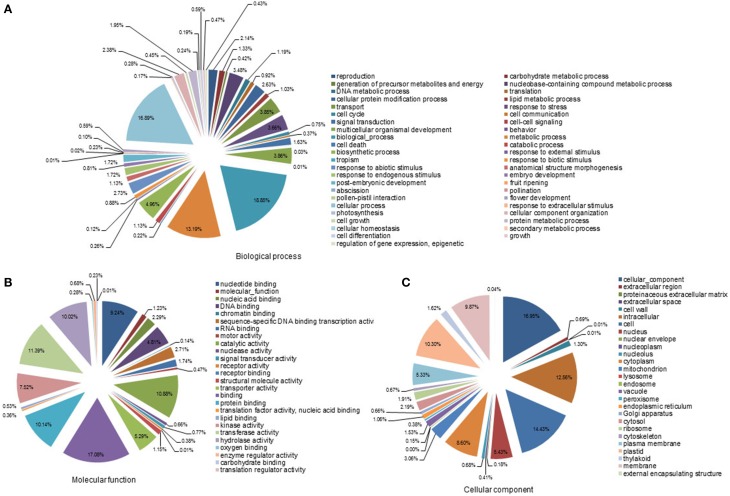
**Gene ontology analyses of all contigs**. SaGI01 contigs were assigned to GO slim terms for biological processes **(A)**, molecular functions **(B)**, and cellular components **(C)**. Numbers indicate percentages of each GO slim term within main ontologies.

For functional annotation of the contigs, a BlastX search was performed against the NCBI non-redundant protein (Nr) database with a cut-off *E* < 10^−15^. Of the 56,011 contigs, 26,626 sequences were annotated as homologs of known proteins. Furthermore, the *S. album* contigs were compared with the genomes of *Vitis vinifera, Populus trichocarpa*, and *Arabidopsis thaliana*. About 54, 53, and 49% of the *S. album* contigs showed a homology hit with genes in these three species, respectively (data not shown). Based on this annotation, the *S. album* contigs were classified into 98 GO slim terms (Figures 1A–C). Classification into cellular component proteins and cell proteins dominated the cellular component category, accounting for about 17 and 14% of the contigs, respectively. The molecular function category was classified into two main groups: binding proteins and enzyme activity proteins. Of these, binding (17.1%), protein binding (10.1%), nucleotide binding (9.2%), transferase activity (11.4%), catalytic activity (10.9%), and hydrolase activity (10.0%) were the most prominent. In the biological process category, 48.9% of contigs were classified into three categories: biological, cellular, and metabolic processes.

### Tissue-specific transcriptome analysis and identification of differentially expressed genes

**Figure 2:**
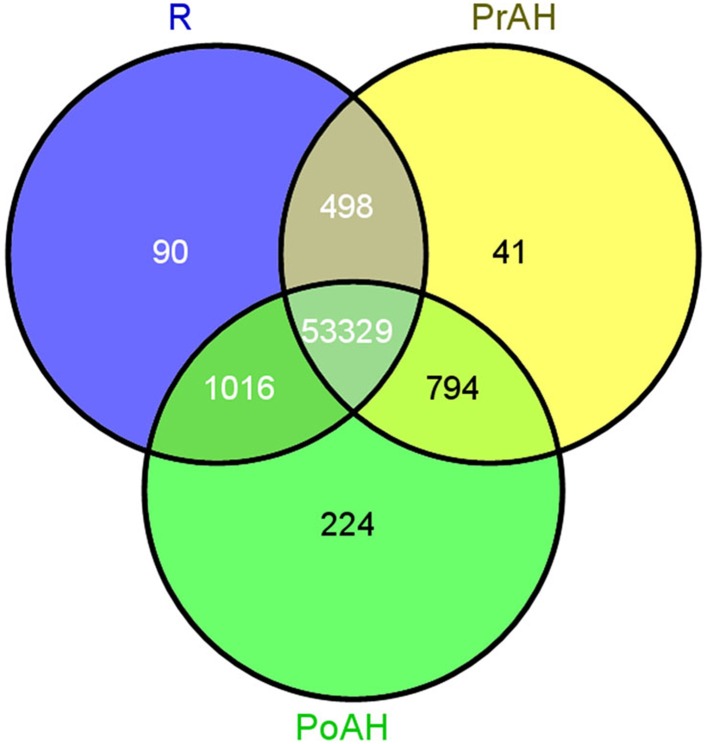
**Venn diagram for tissue-specific expression of the contigs at different stages of haustorial development**. In total 55,992 contigs were expressed above a threshold of FPKM ≥1 in all three tissues. Of these 53,329 were expressed across the three tissues while 355 contigs were preferentially expressed in only one tissue. R, non-haustorial root; PrAH, pre-attachment haustoria; PoAH, post-attachment haustoria.

**Figure 3:**
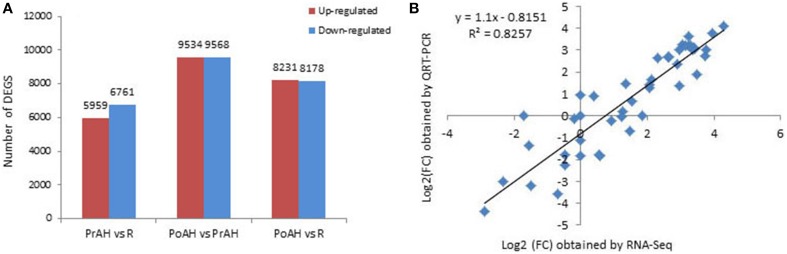
**Analysis of differentially expressed genes (DEGs) during haustorial development**. **(A)** Number of DEGs with an absolute value of |log2 ratio ≥1|among PrAH vs. R, PoAH vs. PrAH, and PoAH vs. R. **(B)** Comparison of the log_2_ (FC) of 24 selected transcripts using RNA-seq and qRT-PCR data.

The initiation and development of the haustorium is a complicated process before it is able to execute its functions in parasitic and hemiparasitic plants. The RNA-seq data was used to assess differences in gene expression involved in the two typical stages of haustorium development, i.e., early haustorial development (PrAH), and early stage of the interaction of *S. album* with the host root (PoAH). The FPKM values were calculated to quantify the expression levels of all contigs. Transcripts with a FPKM value of ≥1 in each sample were comparatively analyzed among R, PrAH, and PoAH. The majority of them (53,329 contigs) were expressed in all three tissues, indicating that haustorial and root development shared a very similar set of genes (Figure 2). A total of 498, 794, and 1016 transcripts were present in R and PrAH, PrAH, and PoAH, and R and PoAH. Only 90, 41, and 224 transcripts were preferentially expressed in the three tissues, respectively. We further identified DEGs with an absolute value of |log2 ratio ≥1|for the three pairwise comparisons (Figure 3A), and validated the reliability of the transcriptomic profiling data by quantifying transcript abundances of 24 DEGs selected using qRT-PCR (Figure 3B).

### Important metabolic pathway analyses during haustorial development

The annotation of functional characteristics performed according to the MapMan functional bin classification showed that approximately 67.7% of these sequences could be categorized into 34 Mapman bins whose percentages of RNA, protein, and signaling were higher than other functions (Figure S3). All DEGs showing any difference in their expression level among the three pairwise comparisons were visualized using the MapMan program by searching against the generated SAGI-Mapman reference file for metabolic pathway analyses (Table S2). These DEGs could be mapped to all 35 MapMan functional categories (Table 2). The most significant of these BINs were related to RNA, signaling, development, and stress in PrAH compared to R, while those in BINs related to RNA, signaling, hormone metabolism, cell wall, development, miscelaneous, mitochondrial electron transport/ATP synthesis, stress, secondary metabolism, and transport significantly dominated PoAH relative to PrAH and R.

**Table 2:** **Functional categorization of differentially expressed genes among the three pairwise comparisons performed with MapMan tool**.

**BIN no**.	**MapMan BIN description**	**No. of elements**	***P*****-value**		
			**PrAH vs. R**	**PoAH vs. PrAH**	**PoAH vs. R**
35	Not assigned	31146	5.61E-20	< 1E-20	< 1E-20
27	RNA	5071	< 1E-20	< 1E-20	1.46E-10
30	Signaling	2504	3.00E-08	< 1E-20	1.72E-12
17	Hormone metabolism	824	0.9	1.40E-19	4.17E-14
10	Cell wall	734	0.99	8.27E-12	8.65E-08
33	Development	1257	1.87E-04	3.30E-11	3.64E-03
26	Misc	1706	1	8.75E-10	1.03E-09
9	Mitochondrial electron transport/ATP synthesis	245	1	1.20E-08	1.10E-12
20	Stress	1270	7.38E-04	4.13E-07	3.20E-01
16	Secondary metabolism	545	0.94	2.75E-06	2.89E-09
34	Transport	1688	1	6.73E-06	2.42E-05
28	DNA	1234	8.05E-03	1.88E-04	0.17
11	Lipid metabolism	606	0.38	1.39E-02	6.11E-04
21	Redox	275	0.45	4.70E-02	0.18
1	PS	250	0.70	4.70E-02	0.3
31	Cell	1627	0.75	0.13	0.3
2	Maior CHO metabolism	177	0.99	0.14	0.32
5	Fermentation	17	0.82	0.25	0.17
3	Minor CHO metabolism	235	0.66	0.31	0.78
14	S-assimilation	14	1	0.66	0.68
23	Nucleotide metabolism	273	1	0.66	0.67
29	Protein	5546	0.94	0.71	0.31
15	Metal handling	89	0.9	0.74	0.15
24	Biodegradation of Xenobiotics	39	0.93	0.74	0.33
32	Micro RNA, natural antisense etc	6	0.99	0.76	0.84
25	C1-metabolism	34	0.99	0.87	0.95
19	Tetrapyrrole synthesis	76	0.87	0.88	0.98
12	N-metabolism	32	0.98	0.92	0.85
13	Amino acid metabolism	419	0.98	0.92	0.63
6	Gluconeogenesis/glyoxylate cycle	13	0.31	0.93	0.47
4	Glycolysis	121	0.99	0.93	0.96
18	Co-factor and vitamine metabolism	141	1	0.95	0.94
22	Polyamine metabolism	27	1	0.95	0.9
7	OPP	36	1	0.99	0.93
8	TCA/org transformation	106	0.99	1	0.86

An overview of changes in transcript abundance associated with primary metabolism showed that many genes associated with cell wall metabolism and mitochondrial respiratory chain function were highly expressed in PrAH vs. R and PoAH vs. PrAH (Figure S4). Most genes encoding pectinesterase (PE), polygalacturonase (PG) precursor, pectin lyase-like superfamily protein (PL), xyloglucan endotransglucosylase/hydrolase (XTH), and expansin-like (EXL) which were associated with cell wall modification and degradation were up-regulated in PrAH and PoAH than in R (Table S4). Moreover, several transcripts were preferentially expressed in PrAH and PoAH, such as β-D-xylosidase (XYL), pectin lyase (PL), and glycosyl hydrolase superfamily protein (GH). These results suggest that cell wall remodeling could be involved in the growth and differentiation of haustoria.

More interestingly, DEGs associated with the mitochondrial respiratory chain function were strongly up-regulated in PrAH and PoAH (Figure S4). A total of 4, 2, 10, and 2 genes encoding Complex I, III, IV, and V of the mitochondrial membrane, respectively, were specific to PrAH, and 2, 2, 3, and 1 gene in the corresponding complexes were unique to PoAH (Table S5). All transcripts encoding cytochrome c oxidases were up-regulated in PoAH vs. PrAH compared to PrAH vs. R. These results suggest that transcripts associated with mitochondrial respiratory functions played important roles in haustorial development and were likely necessary to provide energy for this rapidly growing organ and for establishing host-parasite connections in *S. album*.

The RNA-seq data showed that transcripts associated with protein turn-over were detected during haustorial development. There were 1116, 2381, and 1380 genes classified as being involved in protein synthesis, degradation and modification, respectively, by MapMan analysis of the DEGs (data not shown). We found that genes encoding ribosomal proteins were significantly up-regulated in PoAH compared to PrAH, 20 of which were only expressed in PoAH (Table S6). The number of DEGs associated with protein modification and degradation was higher in PoAH vs. PrAH and PoAH vs. R than in PrAH vs. R. These results indicate that ribosome biogenesis and protein turn-over increased when the haustorium invaded the host root. This is in agreement with the results of histological observations in our previous study (Zhang et al., 2012), in which proteinaceous compounds were abundant during haustorium development, especially in the interactive stage of haustoria and the host root.

### Genes associated with haustorial formation

Many DEGs encoding nodulin-like proteins were detected in the Mapman functional classification associated with plant development, of which some transcripts encoding early nodulin 93 and homologs of *Medicago truncatula*, annotated as MtN3 family proteins, showed higher expression in PrAH and PoAH than in R (Table S7). Moreover, we found many DEGs annotated as GRAS family transcription factor (TF) in the DEGs associated with plant development. Interestingly, the most abundant class of TFs identified as being differentially expressed by the MapMan tool were the GRAS TFs. Of these, 49 (96%) and 11 (30%) were up-regulated in PrAH vs. R and PoAH vs. PrAH, respectively. Some DEGs that were up-regulated four-fold in the PrAH vs. R comparison were specific to haustoria (Table S7). This suggested that GRAS TFs might be critical for haustorium formation in *S. album*.

### Expression analysis of hormone-related genes during haustorial development

Phytohormones function to coordinately regulate plant growth and development. To detect the relationship between phytohormones and haustorial development, a total of 191 DEGs with an absolute value of |log_2_ ratio ≥1|in at least one pairwise comparison of the three analyzed tissues associated with plant hormone biosynthesis, metabolism, and signal transduction pathways were identified in this study, including auxin, CK, GA, ABA, ET, BR, and JA. The largest group consisted of 91 auxin-related DEGs involved in auxin transport and signal transduction (Figure S5).

**Figure 4:**
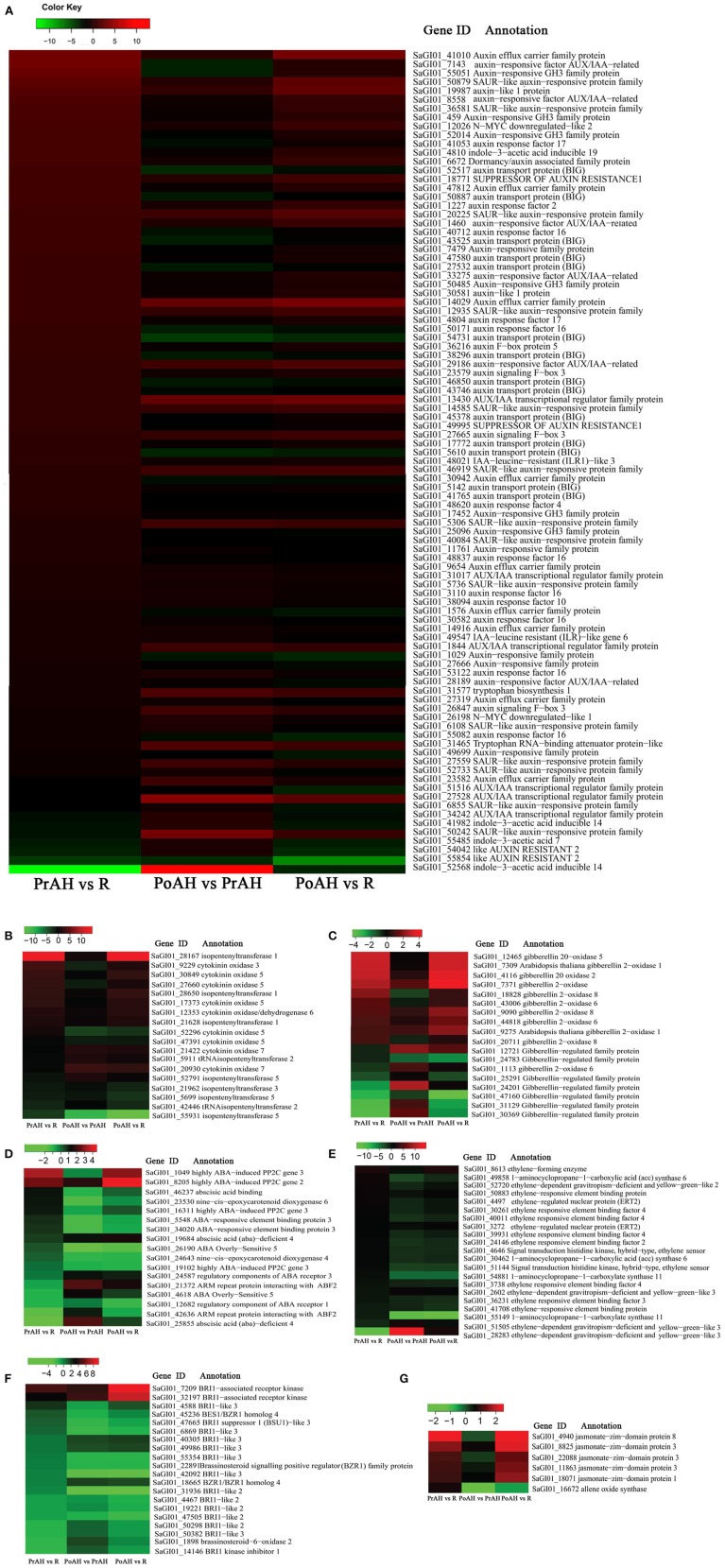
**Heat map diagrams of relative expression levels of DEGs annotated in biosynthesis, catabolism, and signal transduction pathways of auxin (A), CK (B), GA (C), ABA (D), ET (E), BR (F), and JA (G) among the three pairwise comparisons**. The ratios are expressed as log2 FPKM (PrAH/R, PoAH/PrAH, and PoAH/R) values. Red and green colors indicate a relative increase or decrease in expression in the three pairwise comparisons.

**Figure 5:**
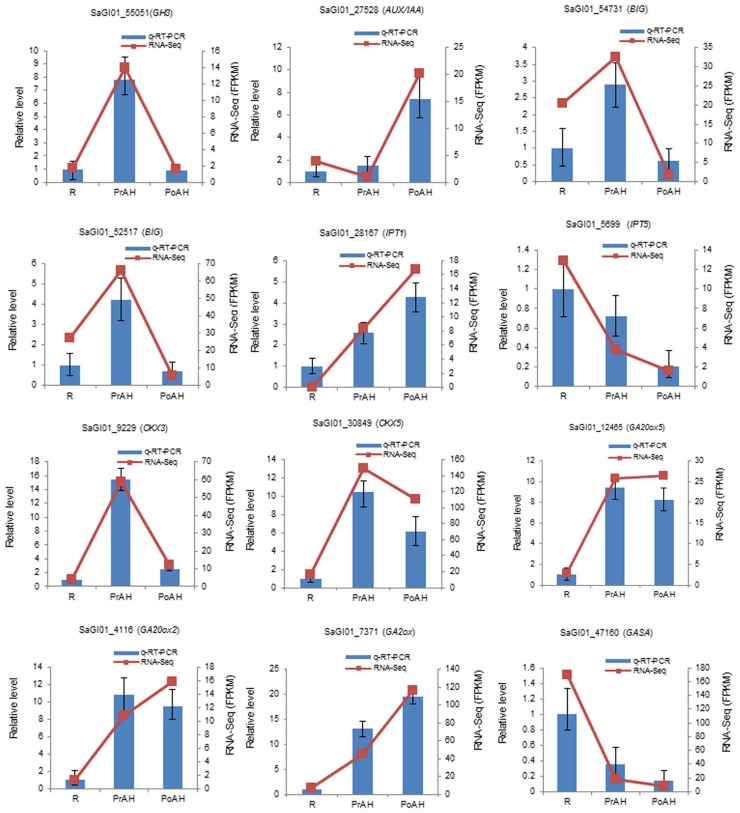
**Expression of selected genes involved in auxin signal transduction, and cytokinin and gibberellin biosynthesis and metabolism quantified by RNA-seq and qRT-PCR**. The left *y*-axis indicates relative expression levels of DEGs calibrated against R. The right *y*-axis indicates FPKM values of genes from RNA-seq data. GH3, GRETCHEN HAGEN; Aux/IAA, auxin/indole-3-acetic acid; BIG, a calossin-like protein; IPT, isopentenyltransferase, CKX, cytokinin oxidase; GA20ox, gibberellin 20-oxidase; GA2ox, gibberellin 2-oxidase; GASA, Gibberellin-regulated family protein.

We constructed heat maps of the DEGs associated with phytohormone-related genes during haustorial development. The majority of auxin-related genes showed higher expression in PrAH vs. R, but many of these genes were down-regulated in PoAH when compared to PrAH (Figure 4A, Table S8). For auxin transporters, three genes annotated as auxin-like 1 (LAX), i.e., auxin influx carriers, and four genes annotated as auxin efflux carriers (AEC) were up-regulated in PrAH when compared to R, and one *AEC* (SaGI01_41010) that showed a 10-fold higher expression in PrAH than in R. Interestingly, all 14 DEGs coding for BIGs, functioning in polar auxin transport (Gil et al., 2001), were up-regulated in PrAH when compared to R, but were down-regulated in the PoAH vs. PrAH comparison, suggesting that an increase in IAA level might be required for haustorial initiation. High auxin levels can trigger the upregulation of auxin-responsive genes, including *auxin response factors* (*ARF*), *Gretchen Hagen 3* (*GH3*), and *small auxin up RNA* (*SAUR*) (Bargmann and Estelle, 2014). In our transcriptome data, we found that four out of six *GH3* genes were up-regulated in PrAH compared to R, but all were down-regulated in PoAH when compared to PrAH. Similarly, some *ARF* genes were up-regulated in PrAH compared to R and all were down-regulated in PoAH vs. PrAH. Some *AUX/IAA* genes, which repress auxin-mediated signaling at a low level of auxin, were up-regulated in PoAH compared to PrAH, one of which was expressed about 16-fold more in PoAH than in PrAH. These results also provide evidence that an increase in IAA concentration occurs during haustorium initiation. The qRT-PCR results for four genes involved in auxin signal transduction and transport were consistent with expression patterns of the RNA-seq data (Figure 5). Taken together, these results indicate that auxin might play a key regulatory role in the initiation of haustoria in *S. album*.

Cytokinin, a major developmental growth regulator, is involved in many aspects of plant development in aerial and subterranean organs. Two major groups of CK biosynthesis and deactivation enzymes were found during haustorial development in this study (Figure 4B, Table S8). Three transcripts encoding for isopentenyltransferase 1 (IPT1), a critical rate-limiting enzyme in CK biosynthesis, were significantly up-regulated in haustoria compared to roots, one of which showed a 200-fold higher expression in haustoria than in roots. The levels of one *IPT2*, one *IPT3*, and two *IPT5* were dramatically down-regulated from root to haustoria, indicating that different CK family members functioned as different regulators in plant organ development. By contrast, the abundance of transcripts encoding cytokinin oxidases (CKX3 and CKX5) involved in CK degradation increased in PrAH compared to R, but the expression levels of these genes decreased in PoAH when compared to PrAH. The expression patterns of four DEGs involved in CK biosynthesis and degradation were confirmed by qRT-PCR (Figure 5).

GAs play a wide range of biological process including seed germination and hypocotyl cell elongation (Ribeiro et al., 2015). The results of our transcriptome analysis showed that DEGs associated with GA biosynthesis and metabolism were significantly up-regulated in haustorial development (Figure 4C, Table S8). *GA20 oxidases 2* and *5* (*GA20ox2* and *GA20ox5*), encoding important rate-limiting enzymes for GA biosynthesis, were expressed about eight-fold more in haustoria than in roots. Four *GA2 oxidases* (*GA2ox*), coding for an enzyme transiting bioactive GAs to inactivation forms of GAs, were also up-regulated in haustoria compared to roots. Of these, one homolog of *Arabidopsis GA2ox 1* showed an eight-fold higher expression in haustoria than in roots. The expression levels of four DEGs were confirmed by qRT-PCR (Figure 5). These results indicate that *GA20ox* and *GA2ox* could coordinately control the steady-state GA level by a dynamic balance between GA synthesis and degradation.

Transcripts involved in ABA biosynthesis and signaling were detected (Figure 4D, Table S8). Two genes encoding 9-*cis*-epoxycarotenoid dioxygenase (NCED), involved in a rate-limiting step in ABA synthesis, were up-regulated in PrAH more than in R. For the ABA signaling pathway, three highly ABA-induced *PP2C2/3* genes (*ABI PP2C*), key negative regulators of ABA signaling, were up-regulated in PrAH more than in R, one of which (SaGI_8205) had eight-fold and 16-fold higher expression in PrAH and PoAH, respectively, than in R. Two positive regulators of ABA responses, coding for ARM repeat protein interacting with ABF2 (ARIA) (SaGI_21372 and SaGI_42636), were down-regulated in PrAH relative to R but were up-regulated in PoAH relative to PrAH. In summary, ABA biosynthesis and signaling were enhanced in haustorial development.

Two genes annotated as 1-aminocyclopropane-1-carboxylic acid (ACC) synthase were up-regulated in PrAH relative to R and PoAH (Figure 4E, Table S8). Transcript abundance of one ethylene-forming enzyme (EFE) gene, which catalyzes the final step of ethylene biosynthesis, was about four-fold higher in PrAH than in R and PoAH. Several genes encoding ethylene-responsive element binding factors and signal transduction histidine kinases had higher expression levels in PrAH than in R and PoAH. These results imply the possible involvement of ethylene in an early stage of haustorial development.

Moreover, BR and JA were also found to be involved in haustorial development (Figures 4F,G, Table S8). In BR signaling pathways, two genes annotated as BRI1-associated receptor kinase (BAK) were highly up-regulated in PrAH and PoAH compared to R. Five of seven DEGs annotated as brassinosteroid insensitive 1-like 3 (BRL3), a close homolog of the BR receptor BRI1, were also up-regulated in PrAH and PoAH more than in R. The expression levels of some downstream genes involved in BR signaling such as *brassinosteroid resistant 1* (*BZR1*) and *BRI1 suppressor 1*-*like 3* (*BSL3*) were up-regulated in PrAH more than in R, but were down-regulated in PoAH relative to PrAH. One allene oxide synthase (AOS) gene, catalyzing the first step in the biosynthesis of JA, was up-regulated in PrAH more than in R. Five genes annotated as jasmonate-ZIM domain (JAZ) proteins were more highly expressed in PrAH and PoAH than in R.

### Hierarchical cluster analysis of differentially expressed genes

**Figure 6:**
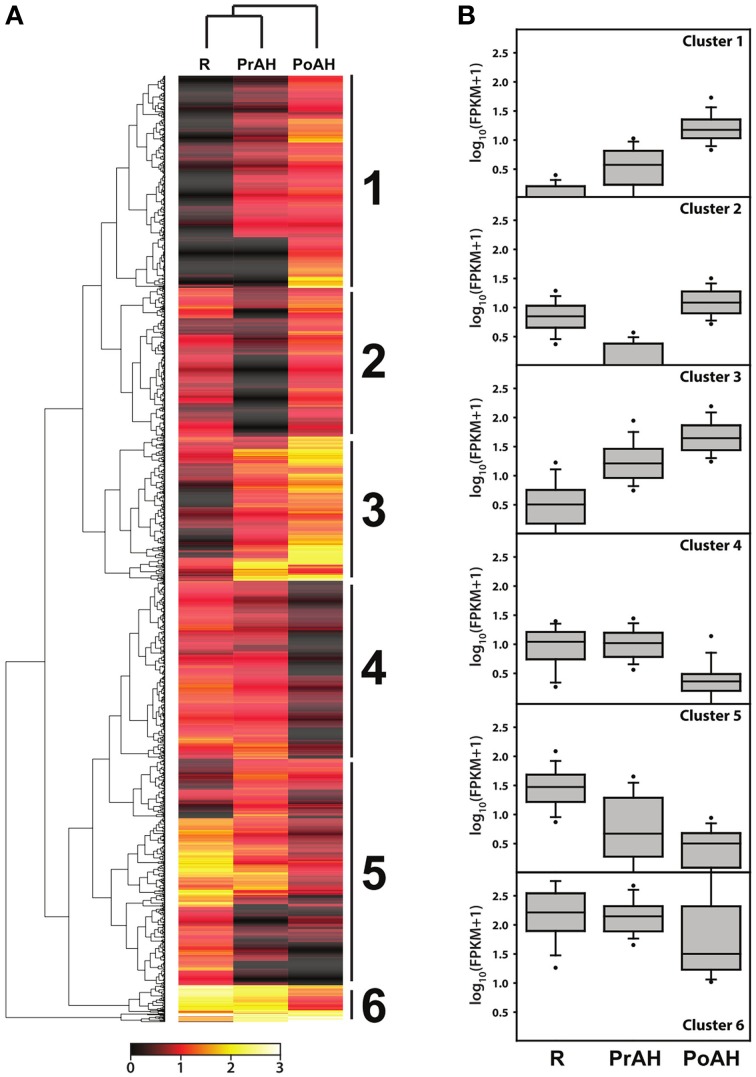
**Hierarchical cluster analyses of differentially expressed genes among R, PrAH, and PoAH**. **(A)** Heat map after hierarchical clustering. **(B)** Box plots for genes in clusters identified in **(A)**.

To gain a better understanding of transcriptional regulation of gene expression involved in haustorial development, a total of 1395 DEGs with a fold change ≥8 in at least one pairwise comparison of the three analyzed tissues were identified in our analysis. A hierarchical cluster analysis was performed to determine the profiles of the DEGs among the three tissues using a Manhattan correlation as the distance metric. Hierarchical clustering showed that these contigs could be divided into six clusters based on the modulation of their expression (Figure 6A). Contigs with the same or similar expression were gathered in the same cluster. Of these, genes in Cluster 1 and 3 had similar patterns of transcript changes, with a low expression in R and increasing expression in PrAH and PoAH (Figure 6B, Table S9), suggesting that these genes were likely involved in haustorial development. We further performed analyses for GO term enrichment of genes in Clusters 1 and 3 (Table 3). GO terms related to oxidation reduction, proteolysis, regulation of catalytic activity, and cell wall modification were significantly enriched in Cluster 1, and GO terms for chemical stimulus, organic substance stimulus, oxidation reduction, response to oxygen levels and hypoxia, and hormone stimulus, especially the response to GA and GA-signaling, were identified in Cluster 3. This indicates that genes associated with mitochondrial oxidation reduction activity, cell wall metabolism, protein metabolism, enzyme catalytic activity, and phytohormone-mediated regulation might play important roles in haustorial development.

**Table 3:** **Gene ontology of significant DEGs distributed in different clusters**.

**Cluster no**.	**GO-ID**	**Description**	**Corrected *p*-value**
1	GO: 8152	Metabolic process	6.05E-06
	GO: 55114	Oxidation reduction	3.55E-05
	GO: 6508	Proteolysis	5.03E-06
	GO: 6412	Translation	3.11E-04
	GO: 50790	Regulation of catalytic activity	1.43E-06
	GO: 65009	Regulation of molecular function	3.62E-06
	GO: 43086	Negative regulation of catalytic activity	2.52E-11
	GO: 44092	Negative regulation of molecular function	1.23E-10
	GO: 6979	Response to oxidative stress	5.13E-04
	GO: 42545	Cell wall modification	8.20E-03
	GO: 45596	Negative regulation of cell differentiation	8.20E-03
	GO: 10214	Seed coat development	8.80E-03
	GO: 43193	Positive regulation of gene-specific transcription	9.37E-03
	GO: 48496	Maintenance of organ identity	2.74E-03
	GO: 48497	Maintenance of floral organ identity	2.74E-03
	GO: 80001	Mucilage extrusion from seed coat	5.32E-03
2	GO: 17004	Cytochrome complex assembly	8.84E-03
	GO: 15886	Heme transport	8.84E-03
	GO: 8535	Respiratory chain complex iv assembly	8.84E-03
3	GO: 0050896	Response to stimulus	1.59E-08
	GO: 0042221	Response to chemical stimulus	7.98E-13
	GO: 00010033	Response to organic substance	1.16E-07
	GO: 00045449	Regulation of transcription	1.45E-03
	GO: 00010556	Regulation of macromolecule biosynthetic process	1.72E-03
	GO: 0031326	Regulation of cellular biosynthetic process	1.81E-03
	GO: 0009889	Regulation of biosynthetic process	1.81E-03
	GO: 0019219	Regulation of nucleobase, nucleoside, nucleotide and nucleic acid metabolic process	1.90E-03
	GO: 0051171	Regulation of nitrogen compound metabolic process	2.08E-03
	GO: 0010468	Regulation of gene expression	3.18E-03
	GO: 0080090	Regulation of primary metabolic process	4.27E-03
	GO: 0060255	Regulation of macromolecule metabolic process	5.70E-03
	GO: 0031323	Regulation of cellular metabolic process	7.03E-03
	GO: 00055114	oxidation reduction	1.28E-05
	GO: 0006355	Regulation of transcription	1.72E-03
	GO: 0051252	Regulation of rna metabolic process	1.72E-03
	GO: 0009719	Response to endogenous stimulus	1.81E-03
	GO: 0009725	Response to hormone stimulus	1.90E-03
	GO: 0009743	Response to carbohydrate stimulus	1.90E-03
	GO: 0010200	Response to chitin	1.72E-03
	GO: 0009739	Response to gibberellin stimulus	8.27E-03
	GO: 0070482	Response to oxygen levels	1.72E-03
	GO: 0001666	Response to hypoxia	1.72E-03
	GO: 0009740	Gibberellic acid-mediated signaling pathway	3.18E-03
	GO: 0071370	Cellular response to gibberellin stimulus	3.18E-03
	GO: 0010476	Gibberellin-mediated signaling pathway	5.26E-03
5	GO: 10033	Response to organic substance	1.73E-03
	GO: 9725	Response to hormone stimulus	8.47E-03
	GO: 48829	Root cap development	1.07E-03
	GO: 10421	Hydrogen peroxide-mediated programmed cell death	8.47E-03
6	GO: 80088	Spermidine hydroxycinnamate conjugate biosynthetic process	3.77E-03

By contrast, the average expression of genes in the other clusters was lower in PrAH and PoAH than in R, with the exception of PoAH in cluster 2. In cluster 2, the expression of all transcripts in PrAH was lowest among the three tissues, but most genes had higher expression in PoAH than in R. A few GO terms were enriched (Table 3) with associated genes related to mitochondria complex assembly. Almost half of the genes in cluster 4 had higher expression in PrAH than in R. These genes can be considered as important candidates for early haustorial development. The expression level of genes in clusters 5 and 6 was highest in R, lower in PrAH and even lower in PoAH. Cluster 6 contained genes that showed high expression levels across all analyzed tissues. Enriched GO terms for genes in Cluster 5 were “root cap development,” “response to hormone stimulus,” and “hydrogen peroxide-mediated programmed cell death” associated with root development.

Taken together, our data identified further transcriptional metabolic pathways that existed in the development of *S. album* haustoria, which included mitochondrial oxidation reduction, cell wall metabolism, protein metabolism, regulation of catalytic activity, and plant hormone-mediated regulation. This again highlights the importance of these processes in haustorial development.

### Genes preferentially expressed in haustoria and roots

Some transcripts preferentially expressed in the three tissues were found. Of these, 19, 48, and 14 genes were annotated in PrAH, PoAH, and R, respectively (Table S10). All annotated transcripts were involved in mitochondria respiratory chains in PrAH, with the exception of one encoding auxin response factor (SaGI01_7143). In PoAH, these preferentially expressed genes mainly encoded 18 ribosomal proteins, eight mitochondria respiratory chains enzymes, four PG precursors, four putative subtilisin homologs, and three Tu elongation factors. Moreover, we detected each gene encoding for glyceraldehyde-3-phosphate dehydrogenase (GAPDH), transporter family protein, phospholipase C (PLC), bifunctional monodehydroascorbate reductase (MDAR) and carbonic anhydrase (CA), ankyrin repeat domain containing protein (Anks), tesmin/TSO1-like CXC domain containing protein (TCX), F-box domain containing protein (FBX), peptidyl-prolyl *cis*-*trans* isomerase (PPIase), desiccation-related protein PCC13-62 precursor (DRP), reticuline oxidase-like protein precursor, and a possible lysine decarboxylase domain-containing protein. In contrast, only five genes coding for ribosomal proteins were observed in R, and the genes encoding hydrolase, ATP synthase subunit β, RNA-dependent RNA polymerase, B12D protein, pectinesterase, nodulin, nodulin MtN3 family protein, transporter family protein, and zinc-finger protein were present. There were significant obvious differences in these preferably genes expressed in these three tissues, implying that haustorial development was markedly different from root development.

## Discussion

High-throughput RNA-seq and *de novo* assembly of RNA-seq data have emerged as powerful and cost-efficient tools for global analyses of transcriptomic changes when no reference genome is available. *S. album* is an economically important plant species for its production of highly valued perfume oils. As a first step for the molecular characterization of oil production in this species, Diaz-Chavez et al. (2013) generated an EST sequence database as a resource and characterized some genes of santalol biosynthesis based on sequence mining. In this study, a transcriptomic analysis of haustorium development in *S. album* was performed using RNA-seq technology to understand metabolic pathways and transcriptional regulatory networks during haustorial development aided by cytomorphological features. A total of 56,011 contigs were generated from a combination of haustoria and seedling root libraries, and nearly half of them could be annotated against the NCBI NR database. To our knowledge, this is the first report of transcriptome analysis of haustorial development in the *S. album*-host interaction.

### Metabolic pathways in haustorial development

The formation of a new organ in plants is closely associated with cell wall modifications. Previous studies revealed that exo-l,4-/β-D-glucosidase appeared in the haustorial region of *C. reflexa*, but could not be detected in its hosts (Nagar et al., 1984). Pectin methylesterase (PME) played an important role in the penetration of intrusive cells into host root tissues in *Orobanche* (Losner-Goshen et al., 1998). Recently, transcriptomic analyses have shown the association of cell-wall modifying β-expansin in the interface of *Triphysaria* with hosts *Zea mays* (maize) or *M. truncatula* and identified genes associated with cell-wall modification during the process of *C. pentagona* parasitism (Honaas et al., 2013; Ranjan et al., 2014). Our results showed that genes associated with cell wall modification and degradation were highly expressed throughout haustorial development. For example, cells of the root cortex became large and loose by the third day of haustorial development (Zhang et al., unpublished) in which genes encoding PE, EXL, and XET were expressed. Cell walls in the host root tissue were degraded by secreted compounds and cell membranes disintegrated in the interface of *S. album* haustoria and the host root (*Kuhnia rosmarnifolia*) (Zhang et al., 2012). Several genes encoding PL, PG, XYL, and GH involved in maceration and degradation of plant cell walls appeared to be differentially expressed in the analyzed stages of haustorial development. Thus, genes associated with cell wall metabolism might play an important role in the invasion of haustoria to establish a physiological bridge connecting with the host.

Early studies showed that a few unidentified proteins were detected in the roots of *S. asiatica* (Wolf and Timko, 1992) and *S. hermonthica* treated with 1 × 10^−5^ M DMBQ (Stranger et al., 1995). More recent research revealed that transcripts involved in protein metabolism were enriched in early haustorial development in the roots of *T. versicolor* (Torres et al., 2005). In our study, both transcriptome data and histological observations revealed that protein turnover was not only involved in early haustorial development, but also was highly active in the stage when haustoria invaded the host root. In several invasive pathogens, transcriptome analysis of haustoria separated from *Golovinomyces orontii* showed that more than half of the 100 most highly expressed genes were involved in protein turnover (Weβling et al., 2012). Proteome analysis of haustoria isolated from barley powdery mildew (*Blumeria graminis*), and wheat leaf rust fungus (*Puccinia triticina*) revealed that most proteins were involved in protein and biological energy metabolism (Godfrey et al., 2009; Song et al., 2011). Together with our data these results collectively imply that active protein metabolism is important for the interaction of haustoria with the host in some parasites and pathogens.

Redox-mediated mechanisms involved in quinone oxidoreductase induced by HIFs are thought to be a trigger of haustorial development in parasitic plants (Matvienko et al., 2001; Bandaranayake et al., 2010, 2012; Ngo et al., 2013). Transcripts associated with mitochondrial respiratory functions were over-represented in the roots of *T. versicolor* when connected to the host or induced by DMBQ within 5 h (Torres et al., 2005). This mechanism was not detected in rice cultivars in response to a susceptible or resistant interaction with *S. hermonthica* (Swarbrick et al., 2008). *S. album* seedlings can generate haustoria without HIFs (Barrett and Fox, 1997). Our study found that mitochondrial respiratory functions could play an important role throughout haustorial development. The majority of ATP in roots is provided by mitochondrial respiration, which is needed for protein synthesis, uptake, transport, and assimilation of minerals, such as phosphate and nitrate (Millar et al., 2011), leading us to believe that mitochondrial respiratory chain functions may be closely related to active protein synthesis during haustorial development and provide energy for haustoria to invade the host root tissue in *S. album*.

### Phytohormone-mediated regulation of haustorial development

Auxin and CK are two classical hormones that regulate many aspects of plant growth and development, and responses to environmental stresses (Müller and Sheen, 2008; Suzaki et al., 2013; de Wit et al., 2014). Auxin and CK have antagonistic effects on lateral root (LR) initiation (Bielach et al., 2012). Auxin plays essential roles in the formation of cluster-roots (CR) and adventitious roots (AR) (Pacurar et al., 2014; Wang et al., 2014). In parasitic plants, the localized accumulation of IAA takes place in early haustorium development in *T. versicolor* (Tomilov et al., 2005). Genes involved in polar auxin transport were also enriched in prehaustoria compared to reference tissues, stem and seedlings of dodder (Ranjan et al., 2014). Our previous study showed that sandalwood haustoria contained very actively dividing meristematic tissue during development and that endogenous IAA and CK levels were higher in haustoria than in roots (Zhang et al., 2012), confirmed by RNA-seq data, in which many auxin and CK-related genes were up-regulated. Polar auxin transport promotes the formation of local auxin maxima and gradients within tissues and further results in patterns of cell division and differentiation in the root meristem (Friml, 2003). The transcriptome data showed that an increase in IAA levels occur during haustoria initiation in sandalwood. This is in agreement with the study by Ranjan et al. (2014), which showed the likely role of auxin in inducing haustoria formation. All these results suggest that haustoria might share a similar initiation pattern of hormone-mediated regulation with root structures such as LR, AR, or CR. This provides a vital clue for artificially increasing the number of haustoria to improve growth by exogenous application of plant growth regulators in beneficial parasitic plants.

Studies have showed that GAs are required for nodule development in legumes, in which two GA biosynthesis genes, *GA20ox*, a key rate-limiting factor controlling the concentration of GAs, and *GA3ox*, catalyzing the final step in the biosynthesis of GAs, are up-regulated at both early and late nodulation (Kouchi et al., 2004; Lievens et al., 2005; Hayashi et al., 2012). GA-biosynthetic enzymes GA2OX8, GA20OX1, GA20OX2, and GA20OX5 were up-regulated in the haustorial stage of dodder and GO terms for GA biosynthetic and metabolic processes were also enriched from the prehaustorial to the haustorial stages (Ranjan et al., 2014). GO terms for the response to GA and the GA-signaling pathway were enriched in haustorial development in this study as also observed in Ranjan et al. (2014)'s study. Probably, GA played a vital role in haustorial development of parasitic plants. Our previous study showed that the endogenous GA_3_ level was high in haustoria (Zhang et al., 2012). Differential gene expression analysis indicates that an optimum level of GA might coordinately control haustorial development in this study. This seems to be similar to nodule development of legumes in which higher or lower concentrations of GA_3_ inhibit nodule formation and development (Ferguson et al., 2005).

A recent study showed that the GO-term for ABA transport was enriched in dodder prehaustoria, stem and seedlings (Ranjan et al., 2014). Our study showed that genes related to ABA biosynthesis and signaling pathway were up-regulated during haustorial development. Indeed, we previously detected an increase in ABA content in sandalwood haustoria (Zhang et al., 2012). Collectively, this suggests that ABA might play a role in the process of haustorial parasitism.

In parasitic plants, the localized accumulation of ET takes place in early haustorium development in *T. versicolor* (Tomilov et al., 2005). In agreement with this finding, our data suggested that an increase in the transcript levels of genes related to ET biosynthesis, such as ACC and EFE, responsive element binding factors and signal transduction histidine kinases, may occur during early stages of haustorial development, indicating a possible result similar to that in *T. versicolor* where ET likely regulates early haustorial development.

The roles of BR and JA in parasitic plants are still not understood. Our data showed that genes associated with BR signaling pathways were expressed. JA regulates plant growth and stress responses (León and Sánchez-Serrano, 1999). There was an increase in the transcript levels of genes related to the JA biosynthesis and signaling pathway during haustorial formation, suggesting the involvement of jasmonate in the process of haustorial development. Besides auxin, CK, GA, and BR are generally considered to be major developmental growth regulators, whereas ABA, ET, and JA are involved in stress responses, but all of these hormones converge on auxin (Wolters and Jürgens, 2009; Depuyd and Hardtke, 2011). Our study supports the involvement of auxin, CK, and GA in haustoria formation based on the transcriptome data, fortified by anatomical features of haustoria (Zhang et al., 2012). However, other hormones such as ABA, ET, BR, and JA are also likely to be involved in haustorial development. In the shoot parasitic weed *C. pentagona*, SL biosynthetic enzymes, MAX1, MAX3, and MAX4 were detected in haustoria more than in prehaustoria, the stem, and seedlings (Ranjan et al., 2014). However, these genes were not observed in our transcriptome data. Probably, there are differences in the regulation of haustorial formation between sandalwood and other parasitic plants which depends on HIFs.

### Specific genes expressed in haustorial tissue

According to this study, tissue-specific genes were found at a higher proportion in PoAH than any other tissues (Figure 2). However, only some of them were annotated (Table S10). Interestingly, almost all of these annotated transcripts were associated with the function of mitochondrial respiratory chains in PrAH, suggesting that mitochondrial respiratory functions could play an important role in early haustorial development. In PoAH, the top two most abundant tissue-specific transcripts encoded ribosomal proteins and mitochondrial respiratory chain enzymes, further highlighting that protein metabolism and mitochondrial functions could play important roles in haustorial development.

Endogenous plant PGs generally work to degrade pectin and soften cell walls in higher angiosperm plants (Fabi et al., 2014). Phytopathogens employ PGs which they secrete to overcome the cell wall's obstruction to acquire nutrients (Oeser et al., 2002; Kalunke et al., 2015). In this study, four preferentially expressed PGs were detected in PoAH. This was consistent with anatomical features in which the cell walls of the host root tissue were degraded by secreted compounds in the interface of haustoria and the host root (Zhang et al., 2012). It is possible that these PGs would allow the parasite to soften host cell walls and penetrate the host root. PGs might play a critical role in the parasite-host interaction in *S. album*. In addition, other genes coding for putative subtilisin homologs, transporter family protein, reticuline oxidase-like protein precursor, Anks, TCX, FBX, GAPDH, PPIase, MDAR, and CA might be involved in the interaction of haustoria and the host. These specific genes provide important information for future studies that would begin to elucidate sandalwood parasitism.

### Developmental control of haustorial formation by nodulins

In previous studies, early nodulation genes (ENODs) and related proteins were found in legume nodule formation (Legocki and Verma, 1979; Nap and Bisseling, 1990) and some of them function mainly in the establishment of symbiosis in legume plants (Scheres et al., 1990). Recent research showed that seven nodulin-like families were also found in non-leguminous plants that are unable to form nodules, such as Arabidopsis, rice, maize or poplar, including *MtN3*/*saliva*/*SWEET, MtN21*/*EamA-like*/*UMAMIT, Early Nodulin-Like, Major Facilitator Superfamily, Sec14p-nodulin* domain proteins, *NOD26-like intrinsic protein* and *Vacuolar iron Transporter*/*nodulin-like* family (Denancé et al., 2014). These nodulin-like proteins are involved in the transport of different solutes, such as nutrients, amino acids, and hormones, but also have a role in plant-microbe interactions (Chen et al., 2012; Ladwig et al., 2012; Streubel et al., 2013). In the last few years, many legume nodule-related or nodule-enhanced genes have been widely investigated (Meschini et al., 2008; Chen et al., 2013). Among them, the GRAS-domain TFs, NSP1, and NSP2, are critical transcriptional regulators in nodule morphogenesis in *M. truncatula* and *L. japonicus* (Kaló et al., 2005; Smit et al., 2005; Heckmann et al., 2006). In our study, some transcripts encoding ENODs, MtN3/saliva/SWEET, and MtN21/EamA-like/UMAMIT were differentially expressed in haustoria when compared to seedling roots. Moreover, the most important group of GRAS TFs was identified during haustorial development. This suggests that these genes might be essential for haustorial morphogenesis in *S. album*. Likely, the molecular mechanism of haustorium formation in the hemiparasite *S. album* shares some similarities with nodule formation in legumes. Elucidation of this interesting question could probably explain the cause of haustorial formation without the need to induce HIFs in the roots of the hemiparasite *S. album*.

## Author contributions

XZ contributed to the design of the research plan, RNA extraction, data analysis and writing of the manuscript. OB contributed to bioinformatic analysis and manuscript revision. JS and JW contributed to manuscript organization, content analysis and revisions. MZ participated in the preparation of experiment materials, RNA extraction, and manuscript revision. GM provided experiment plant material and manuscript revision. JD participated in part of the experimental design and manuscript revision. All authors read and approved the final manuscript and take public responsibility for its content.

### Conflict of interest statement

The authors declare that the research was conducted in the absence of any commercial or financial relationships that could be construed as a potential conflict of interest.

## Footnotes

^1^MapMan Site of Analysis. Available online at: http://mapman.gabipd.org.

## Supplementary material

Figure S1 **Two typical developmental stages of haustoria. (A)**, Pre-attachment haustorium. **(B)**, Post-attachment haustorium. H, haustorium; R, root; PR, parasite root; HR, host root.

Figure S2 **Histogram of sequence length and number of sequences of all contigs**. In total SaGI01 contains 56,011 contigs above a length of 200 bp with an average length of 618 bp.

Figure S3 **MapMan bin classification of SaGI01 contigs**.

Figure S4 **Overview of changes in transcript abundance associated with primary metabolism. (A)** PrAH vs. R and **(B)** PoAH vs. PrAH. Individual genes with an absolute value of |log2 ratio ≥ 1| in the two pairwise comparisons are represented by small squares. Red indicates up-regulation and blue indicates down-regulation of corresponding genes. Large red square indicates up-regulated and down-regulated transcripts involved in mitochondrial electron transport function. Scale on the right depicts the level of expression.

Figure S5 **Statistical analysis of phytohormone-related DEGs among the three pairwise comparisons**. Columns show the number of DEGs involved in auxin, cytokinin (CK), gibberellin (GA), abscisic acid (ABA), ethylene (ET), brassinosteroid (BR), and jasmonic acid (JA) metabolism and signal transduction processes.

Table S1 **MapMan bin data of SaGI01 contigs**.

Table S2 **Differentially expressed genes mapped to the SAGI_Mapman**.

Table S3 **Primer sequences of genes for qRT-PCR**.

Table S4 **MapMan data of DEGs involved in cell wall metabolism**.

Table S5 **Significant DEGs involved in mitochondrial electron transport functions**.

Table S6 **Significant DEGs involved in protein synthesis**.

Table S7 **DEGs associated with plant development**.

Table S8 **Phytohormone-related genes during haustorium development**.

Table S9 **Lists of genes in six clusters. A total of 1395 significantly differentially expressed genes with a fold change ≥ 8 in at least one pairwise comparison of the three analyzed tissues could be divided into six clusters based on modulation of their expression**.

Table S10 **Genes preferentially expressed in haustorial tissues and roots**.
