# Supplementary figures and images for: RNA-Seq analysis identifies key genes associated with haustorial development in the root hemiparasite Santalum album
Source: Front Plant Sci. 2015 Sep 1;6:661. doi: 10.3389/fpls.2015.00661 (PMC4555033; doi:10.3389/fpls.2015.00661)

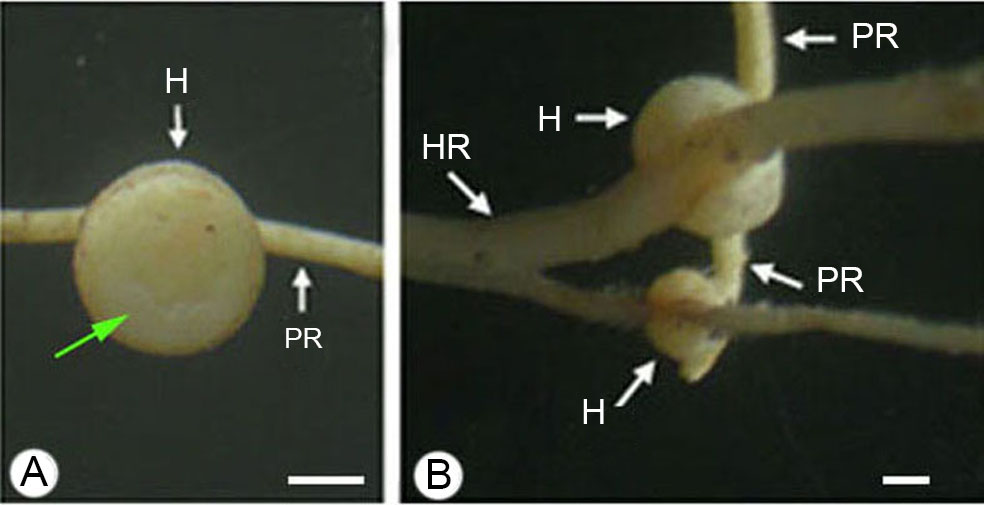

Supplement: Figure S1 — Two typical developmental stages of haustoria. (A), Pre-attachment haustorium. (B), Post-attachment haustorium. H, haustorium; R, root; PR, parasite root; HR, host root. [file Image1.JPEG]

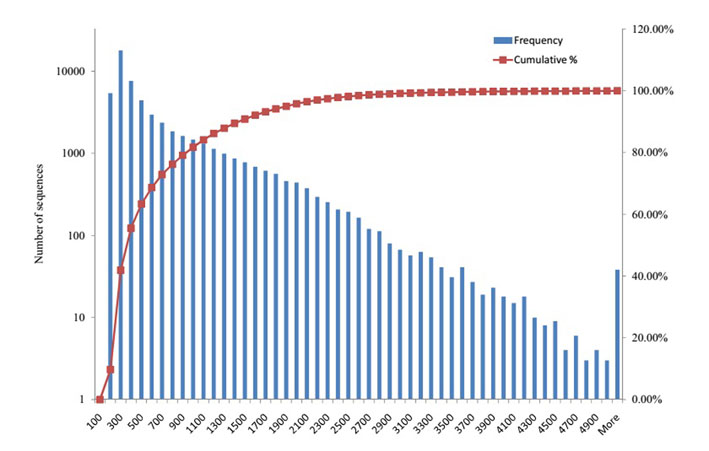

Supplement: Figure S2 — Histogram of sequence length and number of sequences of all contigs. In total SaGI01 contains 56,011 contigs above a length of 200 bp with an average length of 618 bp. [file Image2.JPEG]

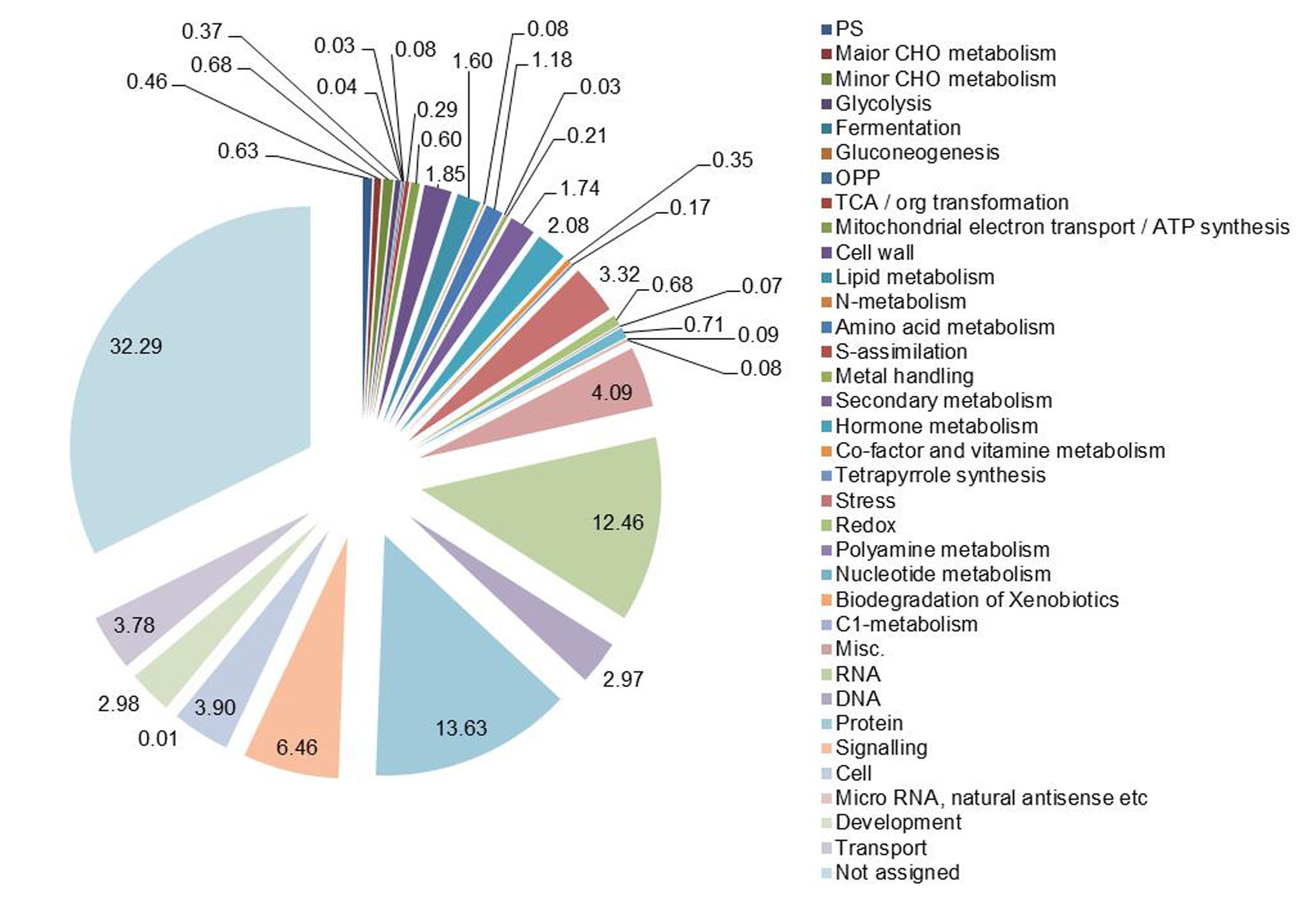

Supplement: Figure S3 — MapMan bin classification of SaGI01 contigs. [file Image3.JPEG]

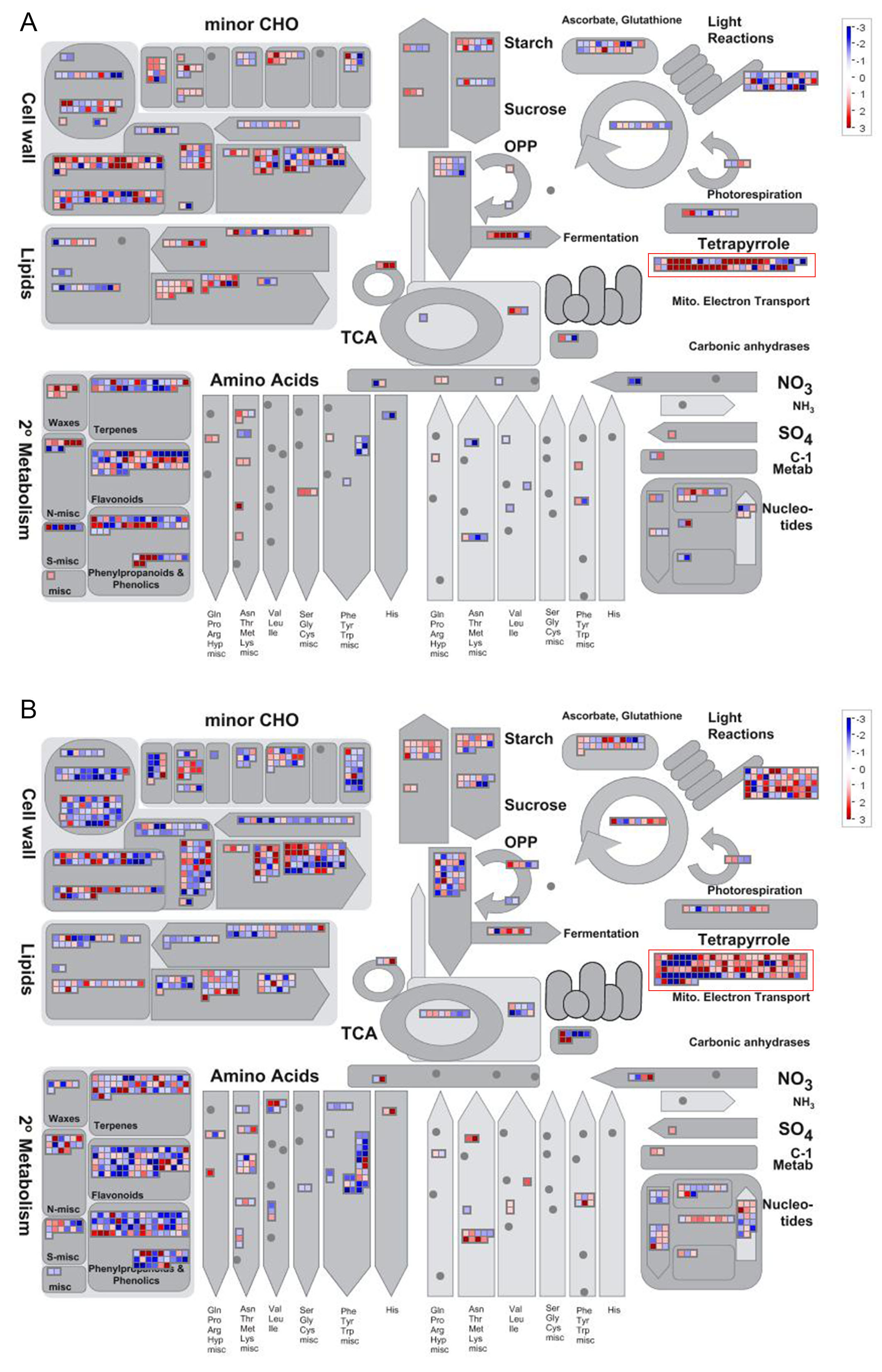

Supplement: Figure S4 — Overview of changes in transcript abundance associated with primary metabolism. (A) PrAH vs. R and (B) PoAH vs. PrAH. Individual genes with an absolute value of |log2 ratio ≥ 1| in the two pairwise comparisons are represented by small squares. Red indicates up-regulation and blue indicates down-regulation of corresponding genes. Large red square indicates up-regulated and down-regulated transcripts involved in mitochondrial electron transport function. Scale on the right depicts the level of expression. [file Image4.JPEG]

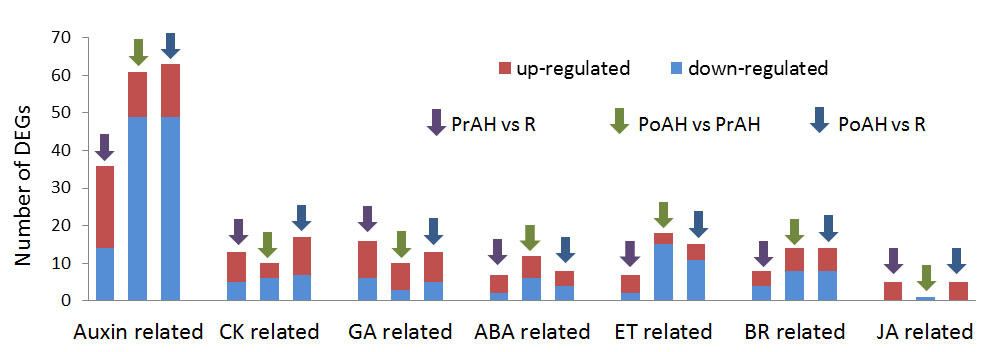

Supplement: Figure S5 — Statistical analysis of phytohormone-related DEGs among the three pairwise comparisons. Columns show the number of DEGs involved in auxin, cytokinin (CK), gibberellin (GA), abscisic acid (ABA), ethylene (ET), brassinosteroid (BR), and jasmonic acid (JA) metabolism and signal transduction processes. [file Image5.JPEG]
